# Secrecy Outage Performance Analysis of Wirelessly Powered IoT System with Randomly Moving Receiving Nodes

**DOI:** 10.3390/s25051386

**Published:** 2025-02-24

**Authors:** Vesna Blagojević, Nadica Kozić, Aleksandra Cvetković, Predrag Ivaniš

**Affiliations:** 1 School of Electrical Engineering, University of Belgrade, 11000 Belgrade, Serbia; predrag.ivanis@etf.rs; 2 Postal Savings Bank, 11000 Belgrade, Serbia; nadica.kozic@posted.co.rs; 3 Faculty of Mechanical Engineering, University of Nis, 18000 Nis, Serbia; aleksandra.cvetkovic@masfak.ni.ac.rs

**Keywords:** mobility model, Nakagami-*m* fading, outage probability, physical layer security, power beacon, random waypoint mobility, secrecy capacity, secrecy outage probability, wireless power transfer

## Abstract

Due to the need for the implementation of various IoT services, novel generation networks are often characterized by a constant requirement for their expansion and a rising number of nodes. The IoT network nodes are usually low power, so security becomes a challenging issue as conventional cryptographic techniques are hard to implement due to power and computational limitations. Besides, wireless power transfer is an appealing approach for powering IoT systems in scenarios where many nodes are placed in hardly accessible areas. Finally, due to a variety of applications, network nodes are often mobile. Motivated by these facts, in this paper, we investigate physical layer security in IoT systems powered by means of a power beacon, where a legitimate user or eavesdropper can be mobile. The closed-form approximate secrecy outage probability expressions are derived for the Nakagami-*m* fading environment and three scenarios of receiving node mobility, described by using a random waypoint model with mobility patterns in one, two or three dimensions. The accuracy of the obtained analytical expressions is corroborated by an independently developed simulation model.

## 1. Introduction

The advanced offer of the high-quality services and conditions needed for their provisioning has made interconnecting in new-generation telecommunication networks very challenging. It is well known that the Internet of Things (IoT) concept has significantly transformed everyday life by introducing various advantages in overall life quality through smart homes, smart cities, smart healthcare services, etc. [1]. Moreover, it has also made possible a huge leap in the quality of industrial process organization, making it very efficient through the minimization or exclusion of human involvement [2], which we often refer to as the Industrial IoT concept and services. Therefore, 5G and the next-generation networks that are currently in the development phase have a task to satisfy a wide set of requirements, some of which include a high level of reliability with extremely high maximal data rates, very low tolerable delay values, etc. At the same time, systems are supposed to provide an extensive network coverage area and an increased level of security [3,4].

It is important to highlight that the IoT concept also contributes to the constantly growing number of nodes and brings novel issues related to their powering, as the traditional approach is impractical when an IoT network is placed in a distant area or has no appropriate energy infrastructure. Furthermore, in many IoT scenarios, conventional battery powering is not practical due to difficulties related to changing batteries and access to numerous nodes. As a potential solution for powering, energy harvesting from existing sources, such as solar, wind, mechanical, etc., has been proposed. However, the drawback of these approaches is that they are highly dependent on weather conditions or other circumstances. Although the idea of wireless energy transfer dates back a long time, it has regained attention recently with technological advancements and maturation. Its basic advantage is that it does not depend on weather conditions, while the same approach can be used for the transmission of information and energy [5,6,7,8]. The potential for energy harvesting has been analyzed in various fading environments, including a generalized type of fading [9]. The harvesting from existing interference sources is investigated in [10], while its impact on cognitive radio systems is analyzed in [11]. In general, in the published literature, two important approaches can be distinguished: the first one corresponds to the simultaneous wireless information and power transfer (SWIPT) [12,13,14], while the other assumes that a dedicated source of power exists, which we refer to as a power beacon (PB) [15,16].

While networks with many devices enable the realization of various advanced services, ensuring security in data transmission represents a complex issue [17]. Due to the low power and limited processing capabilities of IoT devices, implementing conventional cryptographic methods is not feasible [18,19,20,21]. Physical layer security analysis has been attractive for various investigations since the pioneering work of Wyner [22]. Investigations related to covert communication with applied multi-antenna techniques for performance improvement are presented in [23,24]. However, it is important to highlight that most of the published papers are related to the analysis of systems with fixed positions of nodes. Although this analysis is very important, scenarios related to 5G and its successors often encompass cases where users are mobile, leading to both variable small-scale and large-scale fading.

### 1.1. Related Works

User mobility in wireless networks has a significant impact on system performance. Widely accepted models for a user’s random movement are the random direction (RD), which applies a non-uniform spatial distribution [25], and the random waypoint (RWP) model, which is based on uniform spatial distribution [26,27]. The impact of mobility on the system performance when the fading can be described with Nakagami-*m* distribution is provided in [28], while an analysis of the η–μ fading channels is presented in [29]. In both cases, the pattern of the user’s movement is described based on the RWP model with the movement in one, two or three dimensions. A performance analysis of a network using a power beacon powering approach and time-switching protocol with a randomly moving receiver is provided in [30]. A wirelessly powered cognitive radio system with a controlled interference level based on statistical channel state information (CSI) and a randomly moving receiver is analyzed in [16]. The RWP model is used in [31] to investigate the impact on the outage performance of unmanned aerial vehicle (UAV) communications.

However, the physical layer security of the system with a randomly moving receiver is not widely investigated. The secrecy performance of the networks over *N*-Nakagami fading channels has been investigated in [32], which is one of the approaches to model mobile vehicular channels. The impact of mobility on the secrecy performance of the cognitive vehicular network is presented in [33,34], but the used method does not reflect various types of movement. In [35], the impact of the receiver movement on the secrecy capacity and the secrecy outage probability is provided for the Rayleigh fading environment. Further, as wireless powering has been recognized as an important approach in enabling the necessary energy for information transmission, the secrecy performance of wirelessly powered wiretap channels was analyzed in [36]. The secrecy outage analysis of the system with power beacon and transmit antenna selection is provided in [37].

### 1.2. Motivation and Contribution

Wireless power transfer has attracted a lot of attention in scientific literature due to the possibility of providing a practically convenient energy supply. IoT communication networks usually consist of a set of mobile nodes, and the analysis of the impact of the nodes’ mobility is of high importance. Physical layer security is especially important for wirelessly powered networks, as conventional cryptological methods are computationally demanding and impose higher power and processing requirements. However, to the best of the authors’ knowledge, a secrecy performance analysis for the Nakagami-*m* fading environment with randomly moving nodes is not available in the literature, nor is an analysis of the wirelessly powered systems in these propagation conditions. Motivated by this gap, in this paper, we present the secrecy outage analysis of the IoT system that is supplied by means of a dedicated power beacon based on the time-switching (TS) protocol. We analyze the impact of mobility on the secrecy outage probability in cases when either the legitimate user or an eavesdropper is mobile, as well as when both nodes are mobile. The contributions of the paper are as follows:

- We analyze the secrecy performance of a system in which the transmitter is energy-constrained and uses a power beacon for its energy supply for various scenarios of both the legitimate receiver’s and eavesdropper’s movement patterns. For the description of receiver and eavesdropper mobility, the widely accepted RWP model is used;
- By applying the RWP movement model, we have analyzed cases where a legitimate receiver and/or eavesdropper are moving along the line, in the plane within the circle of a given radius or within the sphere, which correspond to the realistic scenarios where a legitimate receiver and eavesdropper can be positioned in various types of ground vehicles or unmanned aerial vehicles;
- The accurate approximation for the secrecy outage probability is derived in the closed form for the following cases: (1) the legitimate receiver is at the fixed position, while the eavesdropper is moving; (2) the legitimate receiver is moving in accordance with the RWP model, while the eavesdropper is at the fixed position and (3) both the legitimate receiver and eavesdropper are moving;
- The analytical approximate expressions are derived for the Nakagami-*m* fading propagation environment and are valid for both integer and non-integer fading parameter values;
- We have developed an independent simulation model of the described system and obtained simulation results that confirmed the high accuracy of the approximate analytical expression for the secrecy outage probability;
- Based on the developed analytical framework and simulation model, we have investigated and discussed the impact of the moving pattern, as well as system and channel parameters, on secrecy performance.

Our results can be used for the security analysis of an IoT system with a wireless power supply, positioned in inaccessible or hazardous areas, where data collecting from IoT sensors can be performed using units mounted on mobile ground or aerial vehicles [38], while communication is endangered by eavesdroppers that can also be mobile.

In the following part, we present an entire analysis in detail. In Section 2, we present the system and channel model for three analyzed scenarios of eavesdropper and legitimate receiver mobility. The secrecy performance analysis is presented in Section 3, where the derivation of novel analytical expressions is provided. The results of the numerical analysis and the corresponding discussion are presented in Section 4. Final considerations and conclusions are given in Section 5.

## 2. System and Channel Model

We consider an IoT system where the transmitter, which is wirelessly powered by means of a power beacon, communicates with the legitimate receiver in the presence of an eavesdropper. The wireless power transfer is performed in accordance with the TS protocol. In this concept, within each time frame of duration *T*, the first part equal to α*T* is dedicated to the power transfer, while the remaining part of the time frame, which is equal to (1 − α)*T*, is used for the transmission of information. The coefficient *α* (0 < *α* < 1) denotes the energy-harvesting time ratio.

The system is situated in an environment where the fading envelope of all links can be modeled by Nakagami-*m* distribution. The distance and the channel envelope between the power beacon and the IoT transmitter are denoted by *D*_1_ and *h*_1_, while the distance and the channel envelope between the IoT transmitter and the legitimate receiver are denoted by *D*_2_ and *h*_2_, respectively. The eavesdropper is placed at a distance *D*_3_ from the IoT transmitter, whereas the corresponding channel envelope is *h*_3_. The path loss exponents in the corresponding links are δ_1_, δ_2_ and δ_3_, respectively.

The signal *x_PB_*, sent from a power beacon with transmitted power *P_PB_*, is represented at the transmitter by the following equation:

(1) $$y_{1}=\sqrt{\frac{P_{PB}}{d_{1}^{\delta_{1}}}}h_{1}x_{PB}+n_{1},$$

where *n*_1_ is the additive white Gaussian noise (AWGN) at the transmitter, while the ratio *d*_1_ = *D*_1_/*D*_0_ represents the distance value normalized to *D*_0_ = 1 m.

**Figure 1:**
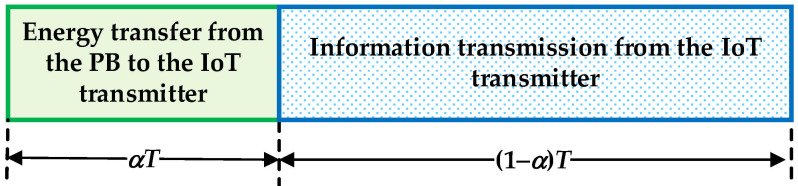
TS protocol for the transmission of information and energy.

As shown in Figure 1, within each time frame of duration *T*, the first part α*T* is dedicated to the energy transfer. Therefore, the harvested energy at the transmitter can be expressed by

(2) $$E_{H}=\eta \alpha T\frac{P_{PB}}{d_{1}^{\delta_{1}}}{\left|h_{1}\right|}^{2},$$

where *η* (0 < *η* < 1) denotes the energy conversion efficiency. As the power of the IoT transmitter depends on the harvested energy and the time during which the signal is transmitted, it is given by the following equation:

(3) $$P_{EH}=\frac{E_{H}}{\left(1-\alpha \right)T}=\frac{\eta \alpha P_{PB}}{\left(1-\alpha \right)d_{1}^{\delta_{1}}}{\left|h_{1}\right|}^{2}.$$

Further, the signal at the legitimate receiver is given as

(4) $$y_{2}=\sqrt{\frac{P_{EH}}{d_{2}^{\delta_{2}}}}h_{2}x_{S}+n_{2},$$

where *n*_2_ is the AWGN at the legitimate receiver with the average value $\sigma_{2}^{2}$, while *d*_2_ = *D*_2_/*D*_0_ is the distance ratio. Then, the signal-to-noise ratio (SNR) at the receiver can be expressed by the following equation:

(5) $$\gamma_{R}=\frac{P_{EH}{\left|h_{2}\right|}^{2}}{d_{2}^{\delta_{2}}\sigma_{2}^{2}}=K_{1}\frac{{\left|h_{1}\right|}^{2}}{d_{1}^{\delta_{1}}}\frac{{\left|h_{2}\right|}^{2}}{d_{2}^{\delta_{2}}},$$

where $K_{1}=\frac{\eta \alpha P_{PB}}{\sigma_{2}^{2}(1-\alpha )}$. By defining variables $\gamma_{1}={\left|h_{1}\right|}^{2}d_{1}^{-\delta_{1}}$ and $\gamma_{2}={\left|h_{2}\right|}^{2}d_{2}^{-\delta_{2}}$, the SNR at the receiver can be written in the following abbreviated form:

(6) $$\gamma_{R}=K_{1}\gamma_{1}\gamma_{2}.$$

We assume that the passive eavesdropper is positioned at the distance $D_{3}$ from the IoT transmitter, so the distance ratio is *d*_3_ = *D*_3_/*D*_0_ and the received signal at the eavesdropper is equal to

(7) $$y_{E}=\sqrt{\frac{P_{EH}}{d_{3}^{\delta_{3}}}}h_{3}x_{S}+n_{3},$$

where *n*_3_ is AWGN at the eavesdropper with the average value $\sigma_{3}^{2}$. We assume that $\sigma_{2}^{2}=\sigma_{3}^{2}$.

Then, the SNR at the eavesdropper can be written as

(8) $$\gamma_{E}=\frac{P_{EH}{\left|h_{3}\right|}^{2}}{d_{3}^{\delta_{3}}\sigma_{3}^{2}}=K_{1}\frac{{\left|h_{1}\right|}^{2}}{d_{1}^{\delta_{1}}}\frac{{\left|h_{3}\right|}^{2}}{d_{3}^{\delta_{3}}},$$

and further presented in the abbreviated form as

(9) $$\gamma_{E}=K_{1}\gamma_{1}\gamma_{3},$$

where $\gamma_{3}={\left|h_{3}\right|}^{2}d_{3}^{-\delta_{3}}$.

In the scenario that we consider, the fading envelope in the channel from the PB to the transmitter follows the Nakagami-*m* distribution, whereas the corresponding channel power gain follows the Gamma distribution. Therefore, the fading distribution in all channels where the receiving node is a non-moving (fixed) one can be described by the following probability density function (PDF):

(10) $$f_{i}\left(x\right)=\frac{1}{\Gamma (m_{i})}{\left(\frac{m_{i}}{\Omega_{i}}\right)}^{m_{i}}x^{m_{i}-1}\exp\left(-\frac{m_{i}}{\Omega_{i}}x\right),i=1,2,3,$$

where $\Omega_{i}=E\left[{\left|h_{i}\right|}^{2}d_{i}^{-\delta_{i}}\right]$ and *m_i_* are Nakagami-*m* parameters of the channel between the PB and the transmitter (*i* = 1), between the IoT transmitter and the legitimate receiver (*i* = 2) and between the transmitter and the eavesdropper (*i* = 3). The mean channel power gain is $\Omega_{i0}=E\left[{\left|h_{i}\right|}^{2}\right]$, for each *i* = 1, 2, 3.

In the case when the receiving node is mobile, we model its movement pattern by a widely accepted RWP mobility model, where it is usually assumed that, depending on the network topology, the nodes’ positions are randomly located within the service area. In the case of a one-dimensional (1D) topology, the receiver is moving along the line with the transmitter or access point being located at the origin. In the case of two-dimensional (2D) topology, the receiver is moving within the circle in one plane, while in three-dimensional (3D) topology, it moves within a spherical space. For cases of both 2D and 3D network topologies, it is assumed that the transmitter is located at the center of the circle (or sphere) [27]. If we define distance *r* between the transmitter and the receiver, the corresponding distributions of nodes’ positions in the RWP model can be described by the PDF of the distance *r*, which is given in the following polynomial form [28,29,30]:

(11) $$f_{r}\left(r\right)=\sum_{i=1}^{n}B_{i}\frac{r^{\beta_{i}}}{D^{\beta_{i}+1}},\;0\leq r\leq D,$$

where *D* is maximal distance, while parameters *n*, $B_{i}$ and $\beta_{i}$ depend on the considered topology, as shown in Table 1.

Due to variable distance between the transmitter and the receiver, as well as the simultaneous fading fluctuation, we have effects of both small- and large-scale fading. Therefore, the PDF of the equivalent channel power gain for the environment with Nakagami-*m* fading fluctuations and RWP moving receiver can be expressed in the closed form by means of incomplete gamma function of the first kind ([39], Equation (8.350.1)) and alternatively in terms of the confluent hypergeometric function _1_*F*_1_(_; _; _) ([39], Equation (9.210.1)), as ([28], Equation (7)):

(12) fjx=mjΩjmjxmj−1Γmj∑i=1nBimjδj+βi+1F11mj+βi+1δj;mj+βi+1δj+1;−mjxΩj,

where *j* = 2, 3, $\Omega_{j}=E\left[{\left|h_{j}\right|}^{2}D_{j,\max}^{-\delta_{j}}/D_{0}^{-\delta_{j}}\right]$ is the average received power at the edge of the coverage area, while $D_{j,\max}$ represents the maximal possible distance between the transmitter and the receiver for the given topology. For *j* = 2, Equation (12) corresponds to the PDF of the channel between the IoT transmitter and the moving receiver, while *j* = 3 corresponds to the PDF of the channel power gain between the IoT transmitter and the moving eavesdropper.

Using the transformation of the confluent hypergeometric function into Meijer G–function ([40], Equation (07.20.26.0006.01)), as

(13) F11mj+βi+1δj;mj+βi+1δj+1;−mjxΩj=mj+βi+1δjG1,21,1mjxΩj1−mj−βi+1δj0,−mj−βi+1δj,

the PDF expression given in (12) can also be written in the form


(14) $$f_{j}\left(x\right)={\left(\frac{m_{j}}{\Omega_{j}}\right)}^{m_{j}}\frac{x^{m_{j}-1}}{\delta_{j}\Gamma \left(m_{j}\right)}\sum_{i=1}^{n}B_{i}G_{1,2}^{1,1}\left(\frac{m_{j}x}{\Omega_{j}}\left|\begin{array}{c} 1-m_{j}-\frac{\beta_{i}+1}{\delta_{j}}\\ 0,-m_{j}-\frac{\beta_{i}+1}{\delta_{j}} \end{array}\right.\right),\;j=2,3.$$

Further, the corresponding cumulative distribution function (CDF) expression can be determined by integrating (14) over variable *x* in the range from 0 to *γ_th_* [41]. By applying ([40], Equation (07.34.21.0084.01)), the final CDF expression is obtained as

(15) $$F_{j}\left(\gamma_{th}\right)={\left(\frac{m_{j}}{\Omega_{j}}\right)}^{m_{j}}\frac{1}{\delta_{j}\Gamma \left(m_{j}\right)}\sum_{i=1}^{n}B_{i}\gamma_{th}^{m_{j}}G_{2,3}^{1,2}\left(\frac{m_{j}\gamma_{th}}{\Omega_{j}}\left|\begin{array}{c} 1-m_{j},1-m_{j}-\frac{\beta_{i}+1}{\delta_{j}}\\ 0,-m_{j}-\frac{\beta_{i}+1}{\delta_{j}},-m_{j} \end{array}\right.\right),\;j=2,3.$$

## 3. Secrecy Performance Analysis

In the following part, we analyze the secrecy outage probability of the wirelessly powered system. To investigate the impact of the node mobility on secrecy performances, we consider scenarios where at least one of the nodes is mobile. We calculate the analytical expression for the secrecy outage that can be presented as the probability that secrecy capacity is below the predetermined limit $R_{S}$, as

(16) $$P_{SOP}=\mathrm{Pr}\left\{C_{S}<R_{S}\right\}.$$

The secrecy capacity is defined as [42]

(17) $$C_{S}=\left\{\begin{array}{ll} C_{R}-C_{E}, & \gamma_{R}>\gamma_{E}\\ 0, & \gamma_{R}\leq \gamma_{E},\; \end{array}\right.$$

where $C_{R}$ is the capacity of the legitimate channel, $C_{E}$ is the capacity of the eavesdropper channel, $\gamma_{R}$ is the SNR at the receiver and $\gamma_{E}$ is the SNR at the eavesdropper.

The capacity of the legitimate channel $C_{R}$ can be obtained as

(18) $$C_{R}=(1-\alpha )\log_{2}(1+\gamma_{R}),$$

where α is the time-splitting factor proportional to the time used for energy transfer, while the capacity of the eavesdropper channel $C_{E}$ can be achieved similarly as

(19) $$C_{E}=(1-\alpha )\log_{2}(1+\gamma_{E}).$$

Using Equations (18) and (19) and replacing them in (17), the secrecy capacity expression can be written in the following form

(20) $$C_{S}=\left\{\begin{array}{ll} (1-\alpha )\log_{2}(1+\gamma_{R})-(1-\alpha )\log_{2}(1+\gamma_{E}), & \gamma_{R}>\gamma_{E},\\ 0, & \gamma_{R}\leq \gamma_{E},\; \end{array}\right.$$

and can be reformulated as

(21) $$C_{S}=\left\{\begin{array}{ll} (1-\alpha )\log_{2}\frac{1+\gamma_{R}}{1+\gamma_{E}}, & \gamma_{R}>\gamma_{E},\\ 0, & \gamma_{R}\leq \gamma_{E}. \end{array}\right.$$

Based on Equation (16) and taking into account that *R_S_* has only non-negative values, the secrecy outage probability expression can be further simplified in the following form:

(22) $$P_{SOP}=\mathrm{Pr}\left\{(1-\alpha )\log_{2}\frac{1+\gamma_{R}}{1+\gamma_{E}}<R_{S}\right\}$$

which is equivalent to

(23) $$P_{SOP}=\mathrm{Pr}\left\{\log_{2}\frac{1+\gamma_{R}}{1+\gamma_{E}}<\frac{R_{S}}{\left(1-\alpha \right)}\right\}.$$

Further, by using the relation $\gamma_{th}=2^{\frac{R_{S}}{1-\alpha }}$ and replacing it in Equation (23), the expression for the secrecy outage probability can be represented as

(24) $$P_{SOP}=\mathrm{Pr}\left\{\frac{1+\gamma_{R}}{1+\gamma_{E}}<\gamma_{th}\right\}.$$

Due to the complex form of the joint PDF of the random variables $\gamma_{R}$ and $\gamma_{E}$ in the case of the Nakagami-*m* fading environment, wireless power transfer and the distribution of link lengths in accordance with RWP moving patterns, the expression for the secrecy outage probability cannot be determined in a mathematically closed form. However, by introducing the approximation [43]

(25) $$\frac{1+\gamma_{R}}{1+\gamma_{E}}\approx \frac{\gamma_{R}}{\gamma_{E}},$$

which represents the lower performance bound, it is possible to obtain the secrecy outage probability closed-form approximation. Based on a certain expression for the lower limit, the influence of system parameters on performance can be analyzed with satisfactory accuracy.

Based on the described approach, the expression for the lower bound of the secrecy outage probability can be written in the form

(26) $$\begin{array}{cc} P_{SOP,LB} & =\mathrm{Pr}\left\{\frac{\gamma_{R}}{\gamma_{E}}\leq \gamma_{th}\right\}=\mathrm{Pr}\left\{\frac{\gamma_{2}}{\gamma_{3}}\leq \gamma_{th}\right\}=\mathrm{Pr}\left\{\gamma_{2}\leq \gamma_{th}\gamma_{3}\right\}\\ & =\int_{\gamma_{3}=0}^{\infty }\int_{\gamma_{2}=0}^{\gamma_{th}\gamma_{3}}f_{2}\left(\gamma_{2}\right)f_{3}\left(\gamma_{3}\right)d\gamma_{2}d\gamma_{3}, \end{array}$$

where *f*_2_(*γ*_2_) and *f*_3_(*γ*_3_) are the PDFs of the channel power gain of the link from the IoT transmitter to the legitimated receiver and from the IoT transmitter to the eavesdropper node, respectively. The expression for the lower bound of the secrecy outage probability can be further written by using the definition of the CDF, in the following form:

(27) $$P_{SOP,LB}=\int_{\gamma_{3}=0}^{\infty }F_{2}\left(\gamma_{th}\gamma_{3}\right)f_{3}\left(\gamma_{3}\right)d\gamma_{3},$$

where $F_{2}\left(\gamma_{th}\right)$ represents the CDF of the channel power gain in the link from the IoT transmitter to the legitimated receiver.

In the following part, we analyze the secrecy performance of a wirelessly powered IoT system, where an IoT node at a fixed position transmits information to the receiver in the presence of the passive eavesdropper for three important scenarios. In the first scenario, the legitimate receiver is mobile, while the eavesdropper is situated at a fixed position. In the second scenario, the legitimate receiver has a fixed position, whereas the eavesdropper is mobile. In the third scenario, an IoT transmitter has a fixed position, whereas both the legitimate receiver and eavesdropper represent moving nodes. For each of the described scenarios, we derive the secrecy outage probability expression.

### 3.1. Scenario 1: The Legitimate Receiver Is Mobile, the Eavesdropper Is Fixed-Positioned

**Figure 2:**
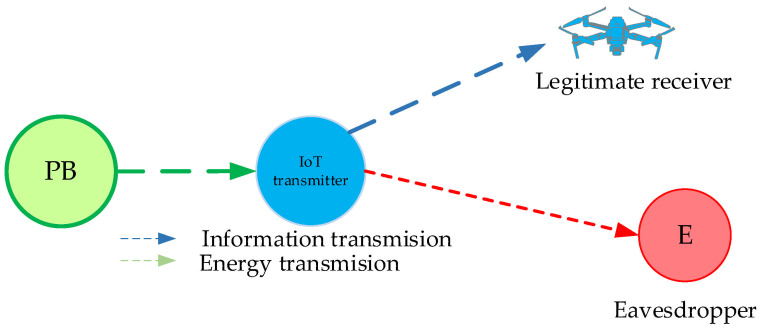
System model in which the legitimate user is mobile and the eavesdropper has a fixed position.

In this scenario, due to the movement of the legitimate receiver, the distance from the fixed IoT transmitter is variable (Figure 2) and the corresponding PDF can be described with Equation (14). For the calculation of approximate expression for the secrecy outage probability defined by Equation (27), we use CDF given by Equation (15) for *i* = 2 and PDF given by Equation (10) for *i* = 3 and obtain

(28) $$\begin{array}{cc} P_{SOP,LB}= & {\left(\frac{m_{2}\gamma_{th}}{\Omega_{2}}\right)}^{m_{2}}{\left(\frac{m_{3}}{\Omega_{3}}\right)}^{m_{3}}\frac{1}{\delta_{2}\Gamma \left(m_{2}\right)\Gamma (m_{3})}\sum_{i=1}^{n}B_{i}\times \\ & \int_{\gamma_{3}=0}^{\infty }\gamma_{3}^{m_{2}+m_{3}-1}G_{2,3}^{1,2}\left(\frac{m_{2}\gamma_{th}\gamma_{3}}{\Omega_{2}}\left|\begin{array}{c} 1-m_{2},1-m_{2}-\frac{\beta_{i}+1}{\delta_{2}}\\ 0,-m_{2}-\frac{\beta_{i}+1}{\delta_{2}},-m_{2} \end{array}\right.\right)\exp\left(-\frac{m_{3}}{\Omega_{3}}\gamma_{3}\right)d\gamma_{3}, \end{array}$$

where $\Omega_{2}=\Omega_{20}D_{2,\max}^{-\delta_{2}}/D_{0}^{-\delta_{2}}$, while *D*_2,max_ is the maximal distance between the IoT transmitter and the legitimate receiver.

By transforming the exponential function into the Meijer G–function ([40], Equation (01.03.26.0004.01)) as

(29) $$\exp\left(-\frac{m_{3}}{\Omega_{3}}\gamma_{3}\right)=G_{0,1}^{1,0}\left(\frac{m_{3}}{\Omega_{3}}\gamma_{3}\left|\begin{array}{c} -\\ 0 \end{array}\right.\right),$$

and further by replacing in Equation (28) and using ([40], Equation (07.34.21.0011.01)), the expression (28) can be finally written in the following closed form:

(30) $$\begin{array}{l} P_{SOP,LB}={\left(\frac{m_{3}\Omega_{2}}{m_{2}\gamma_{th}\Omega_{3}}\right)}^{m_{3}}\frac{1}{\delta_{2}\Gamma \left(m_{2}\right)\Gamma (m_{3})}\times \\ \;\;\;\;\sum_{i=1}^{n}B_{i}\cdot G_{3,3}^{3,1}\left(\frac{m_{3}\Omega_{2}}{m_{2}\gamma_{th}\Omega_{3}}\left|\begin{array}{c} 1-m_{2}-m_{3},1-m_{3}+\frac{\beta_{i}+1}{\delta_{2}},1-m_{3}\\ 0,-m_{3},\frac{\beta_{i}+1}{\delta_{2}},-m_{3} \end{array}\right.\right). \end{array}$$

### 3.2. Scenario 2: The Legitimate Receiver Is Fixed-Positioned, the Eavesdropper Is Mobile

**Figure 3:**
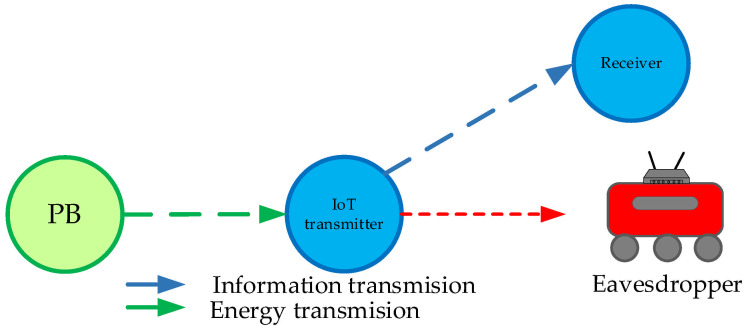
System model in which the legitimate user has a fixed position and the eavesdropper is mobile.

In this scenario, given in Figure 3, the legitimate receiver is positioned at a fixed location, while the distance between the IoT transmitter and the eavesdropper is variable with the maximal distance $D_{3,\max}$. We obtain an approximate expression for secrecy outage probability by applying the PDF given by Equation (14) for the Nakagami-*m* fading and RWP moving node for *i* = 3 and the CDF for Nakagami-*m* fading in the link with a constant distance between the IoT transmitter and the receiver. Then, the CDF expression for the SNR in the legitimate link is presented in the form of a Meijer G–function by using the transformation of exponential into the Meijer G–function ([40], Equation (01.03.26.0004.01)), where we obtain

(31) $$\begin{array}{cc} F_{2}\left(\gamma_{th}\right) & =\frac{1}{\Gamma (m_{2})}{\left(\frac{m_{2}}{\Omega_{2}}\right)}^{m_{2}}\int_{0}^{\gamma_{th}}x^{m_{2}-1}\exp\left(-\frac{m_{2}}{\Omega_{2}}x\right)dx=\\ & =\frac{1}{\Gamma (m_{2})}{\left(\frac{m_{2}}{\Omega_{2}}\right)}^{m_{2}}\int_{0}^{\gamma_{th}}x^{m_{2}-1}G_{0,1}^{1,0}\left(\frac{m_{2}x}{\Omega_{2}}\left|\begin{array}{c} -\\ 0 \end{array}\right.\right)dx. \end{array}$$

After applying ([40], Equation (07.34.21.0084.01)), we get

(32) $$F_{2}\left(\gamma_{th}\right)=\frac{1}{\Gamma (m_{2})}{\left(\frac{m_{2}\gamma_{th}}{\Omega_{2}}\right)}^{m_{2}}G_{1,2}^{1,1}\left(\frac{m_{2}\gamma_{th}}{\Omega_{2}}\left|\begin{array}{c} 1-m_{2}\\ 0,-m_{2} \end{array}\right.\right).$$

Finally, the secrecy outage probability expression (27) becomes

(33) $$\begin{array}{c} P_{SOP,LB}=\frac{1}{\delta_{3}\Gamma (m_{2})\Gamma \left(m_{3}\right)}{\left(\frac{m_{2}\gamma_{th}}{\Omega_{2}}\right)}^{m_{2}}{\left(\frac{m_{3}}{\Omega_{3}}\right)}^{m_{3}}\sum_{i=1}^{n}B_{i}\times \\ \;\;\;\;\;\;\;\;\;\;\;\;\int_{0}^{\infty }\gamma_{3}^{m_{2}+m_{3}-1}G_{1,2}^{1,1}\left(\frac{m_{2}\gamma_{th}\gamma_{3}}{\Omega_{2}}\left|\begin{array}{c} 1-m_{2}\\ 0,-m_{2} \end{array}\right.\right)G_{1,2}^{1,1}\left(\frac{m_{3}\gamma_{3}}{\Omega_{3}}\left|\begin{array}{c} 1-m_{3}-\frac{\beta_{i}+1}{\delta_{3}}\\ 0,-m_{3}-\frac{\beta_{i}+1}{\delta_{3}} \end{array}\right.\right)d\gamma_{3}, \end{array}$$

and the final closed form is obtained by using ([40], Equation (07.34.21.0011.01)) as

(34) $$\begin{array}{l} P_{SOP,LB}=\frac{1}{\delta_{3}\Gamma (m_{2})\Gamma \left(m_{3}\right)}{\left(\frac{m_{3}\Omega_{2}}{\Omega_{3}m_{2}\gamma_{th}}\right)}^{m_{3}}\times \\ \;\;\;\;\sum_{i=1}^{n}B_{i}G_{3,3}^{2,2}\left(\frac{m_{3}\Omega_{2}}{\Omega_{3}m_{2}\gamma_{th}}\left|\begin{array}{c} 1-m_{3}-\frac{\beta_{i}+1}{\delta_{3}},1-m_{2}-m_{3},1-m_{3}\\ 0,-m_{3},-m_{3}-\frac{\beta_{i}+1}{\delta_{3}} \end{array}\right.\right). \end{array}$$

### 3.3. Scenario 3: Both Legitimate Receiver and Eavesdropper Are Mobile

**Figure 4:**
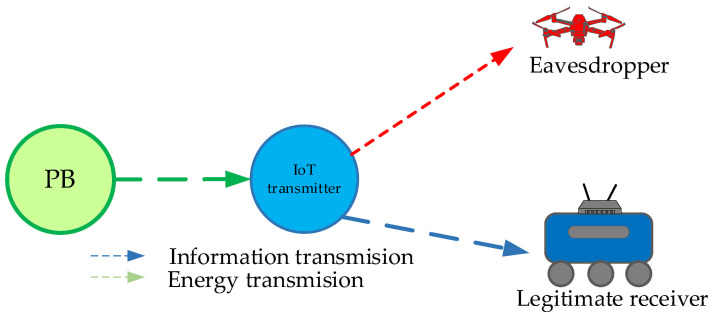
System model in which both legitimate user and eavesdropper are mobile.

When both the legitimate receiver and eavesdropper are moving with respect to the IoT transmitter that sends information, the maximal distance from the IoT transmitter to the legitimate receiver is *D*_2,max_, while the maximal distance to the eavesdropper is *D*_3,max_. This scenario is illustrated in Figure 4. The secrecy outage probability expression is calculated by substituting the CDF expression (15) for *i* = 2 and the PDF expression (14) for *i* = 3 in Equation (27), where we obtain

(35) $$\begin{array}{l} P_{SOP,LB}=\frac{1}{\delta_{2}\delta_{3}\Gamma (m_{2})\Gamma \left(m_{3}\right)}{\left(\frac{m_{2}\gamma_{th}}{\Omega_{2}}\right)}^{m_{2}}{\left(\frac{m_{3}}{\Omega_{3}}\right)}^{m_{3}}\sum_{i=1}^{n}\sum_{j=1}^{n}B_{i}B_{j}\times \\ \;\;\;\;\int_{0}^{\infty }\gamma_{3}^{m_{2}+m_{3}-1}G_{2,3}^{1,2}\left(\frac{m_{2}\gamma_{th}\gamma_{3}}{\Omega_{2}}\left|\begin{array}{c} 1-m_{2},1-m_{2}-\frac{\beta_{i}+1}{\delta_{2}}\\ 0,-m_{2}-\frac{\beta_{i}+1}{\delta_{2}},-m_{2} \end{array}\right.\right)G_{1,2}^{1,1}\left(\frac{m_{3}\gamma_{3}}{\Omega_{3}}\left|\begin{array}{c} 1-m_{3}-\frac{\beta_{j}+1}{\delta_{3}}\\ 0,-m_{3}-\frac{\beta_{j}+1}{\delta_{3}} \end{array}\right.\right)d\gamma_{3}. \end{array}$$

Further, by applying ([40], Equation (07.34.21.0011.01)), the secrecy outage probability can be expressed in the following final form:

(36) $$\begin{array}{c} P_{SOP,LB}=\frac{1}{\delta_{2}\delta_{3}\Gamma (m_{2})\Gamma \left(m_{3}\right)}{\left(\frac{m_{3}\Omega_{2}}{\Omega_{3}m_{2}\gamma_{th}}\right)}^{m_{3}}\sum_{i=1}^{n}\sum_{j=1}^{n}B_{i}B_{j}\times \\ \;\;\;\;\;\;\;\;\;\;\;\;G_{4,4}^{3,2}\left(\frac{m_{3}\Omega_{2}}{\Omega_{3}m_{2}\gamma_{th}}\left|\begin{array}{c} 1-m_{3}-\frac{\beta_{j}+1}{\delta_{3}},1-m_{2}-m_{3},1-m_{3}+\frac{\beta_{i}+1}{\delta_{2}},1-m_{3}\\ 0,-m_{3},-m_{3}+\frac{\beta_{i}+1}{\delta_{2}},-m_{3}-\frac{\beta_{j}+1}{\delta_{3}} \end{array}\right.\right). \end{array}$$

## 4. Numerical Results

In this section, we provide numerical results based on the previously presented analysis. The accuracy of the derived analytical expressions is corroborated by the developed independent Monte Carlo simulation method, which is implemented in MATLAB 2023a [44]. The simulation results are calculated based on the rejection/acceptance technique described in [41], where we have generated a temporally uncorrelated time series with Nakagami-*m* distribution of length *L* = 10^6^ samples. Combining [45,46], we have obtained a statistically accurate simulation method, valid for non-integer values of fading parameter *m*, which represents a special case of the more complex simulation method that we originally proposed in [47]. The numerical results are presented for various channel and system parameters. In all cases, results obtained by using developed simulation models are compared to the results obtained by approximate closed-form expressions given by Equations (30), (34) and (36).

In all simulations, the efficiency coefficient is set to *η* = 0.9, the data rate is *R_S_* = 1 b/s/Hz, the distance between the power beacon and the IoT transmitter is 1 m and the transmit power of the power beacon is 20 dB.

**Figure 5:**
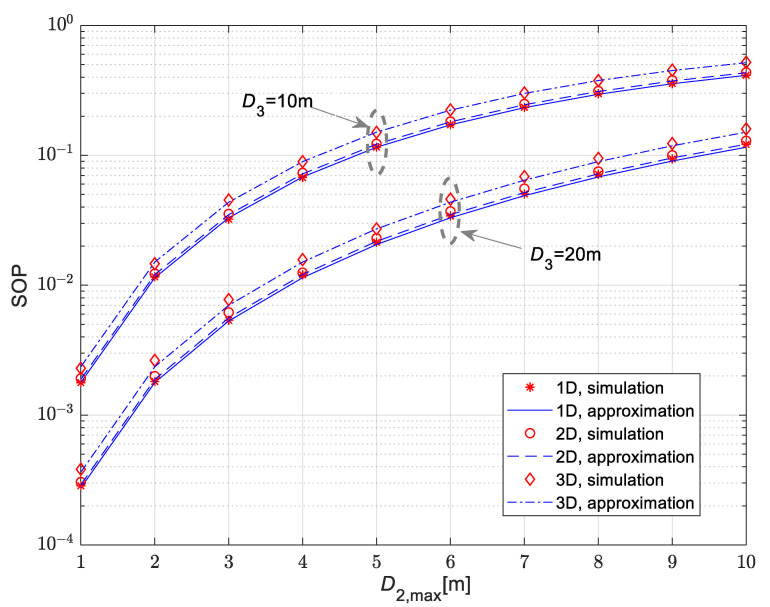
Secrecy outage probability for various movement patterns of the mobile legitimate receiver for various fixed distances of eavesdropper from the IoT transmitter.

**Figure 6:**
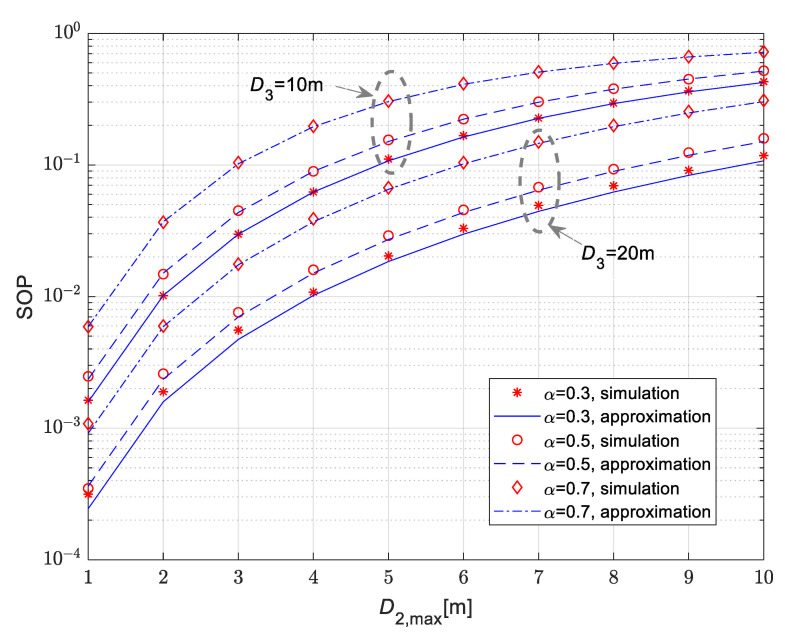
Secrecy outage probability vs. maximal distance of the mobile legitimate receiver for various time-switching coefficients α.

Figure 5 and Figure 6 show the secrecy outage probability for the scenario when the legitimate receiver is mobile, and its movement is modeled by the RWP model. The mean values of channel power gain are $\Omega_{10}=10\;\mathrm{dB}$, $\Omega_{20}=\Omega_{30}=20\;\mathrm{dB}$. The Nakagami-*m* fading parameters in all channels are $m_{1}=m_{2}=m_{3}=2$, while path loss coefficients are δ_1_ = δ_2_ = δ_3_ = 2.7.

The results in Figure 5 are presented for the time-switching coefficient equal to α = 0.5. For the movement of the legitimate receiver, the 1D, 2D and 3D patterns of the RWP model are investigated. According to the obtained results, the secrecy outage probability values are lowest in the case when the receiver is moving along the line, which corresponds to the 1D pattern, while the highest values are obtained in the case of a 3D movement pattern within the sphere of the given radius. For all considered scenarios, the secrecy outage probability increases with the rise of the maximal distance between the IoT transmitter and the legitimate user. Furthermore, the secrecy outage probability values are lower in the case when the eavesdropper is set to a larger distance from the IoT transmitter (it is smaller in the case $D_{3}=20\;m$ than in the case $D_{3}=10\;m$). The dependence of the secrecy outage probability on the time-switching coefficient α is presented in Figure 6, for the case of a mobile legitimate receiver and a fixed-position eavesdropper. In accordance with the expectations, the secrecy outage probability increases for larger values of α. Obtained results presented in both Figure 5 and Figure 6 demonstrate the accuracy of the derived approximation for secrecy outage probability given by closed-form expression (30).

**Figure 7:**
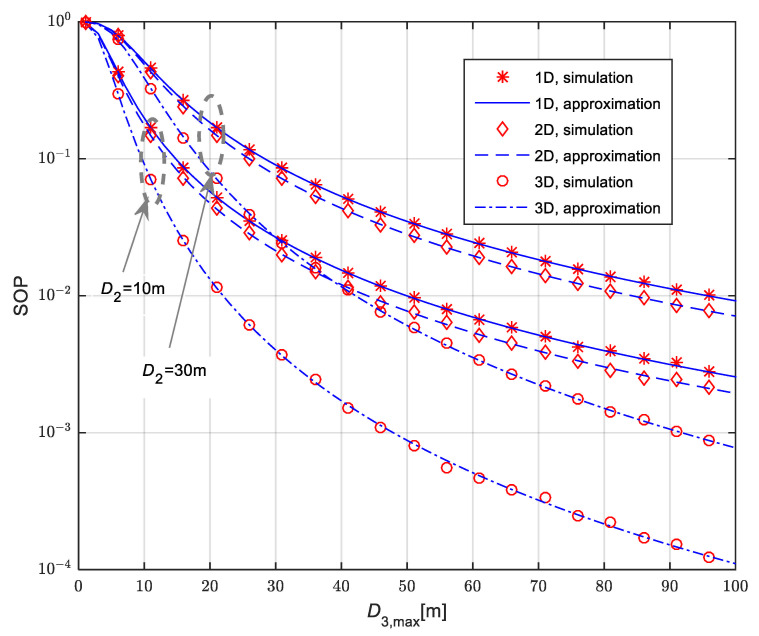
Secrecy outage probability in the case of fixed legitimate receiver for various movement patterns of mobile eavesdropper.

In Figure 7, we present the secrecy outage probability for the scenario when the legitimate receiver is placed at the fixed position, which is equal to $D_{2}=10\;m$ and $D_{2}=30\;m$. The eavesdropper is moving in accordance with the RWP model. The mean values of channel power gain are $\Omega_{10}=20\;\mathrm{dB}$, $\Omega_{20}=20\;\mathrm{dB}$ and $\Omega_{30}=10\;\mathrm{dB}$, while the corresponding Nakagami-*m* non-integer fading parameters values are $m_{1}=2.1$, $m_{2}=3.8$ and $m_{3}=1.2$. The path-loss coefficients have the values of $\delta_{1}=2.1$, $\delta_{2}=1.6$ and $\delta_{3}=2.7$. The time-switching coefficient is equal to α = 0.5. For all analyzed cases, secrecy outage probability is lower in the case when the legitimate receiver is placed at the fixed position with distance $D_{2}=10\;m$ from the IoT transmitter than in the case when it is placed at the distance $D_{2}=30\;m$. As the eavesdropper is moving in accordance with the RWP model in 1D, 2D and 3D areas, the secrecy outage probability is highest in the case of eavesdropper movement in the 1D line and lowest in the case when it moves within the spherical 3D space. Furthermore, with the increase of the maximal distance from the IoT transmitter to the eavesdropper $D_{3,\max}$, the secrecy outage probability values decrease.

**Figure 8:**
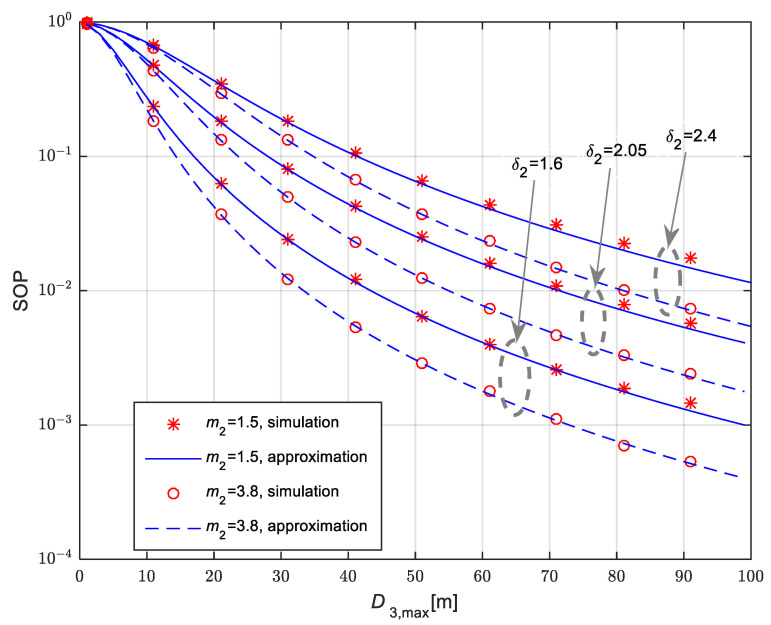
Secrecy outage probability in the case of fixed legitimate receiver for 3D movement of mobile eavesdropper and various fading parameters and path loss exponent values.

Secrecy outage probability for the scenario when the legitimate receiver is placed at the fixed position equal to $D_{2}=20\;m$ is presented in Figure 8. The eavesdropper is moving in accordance with the 3D RWP model. The mean values of channel power gain are $\Omega_{10}=20\;\mathrm{dB}$, $\Omega_{20}=20\;\mathrm{dB}$ and $\Omega_{30}=10\;\mathrm{dB}$. The Nakagami-*m* fading parameters have non-integer values equal to $m_{1}=2.1$ and $m_{3}=0.6$, while the fading parameter in the link to the legitimate receiver is $m_{2}=1.5$ and $m_{2}=3.8$. Secrecy performances are analyzed for three different values of path-loss coefficient in the link to the legitimate receiver equaling $\delta_{2}=24$, $\delta_{2}=2.05$ and *δ*_2_ = 1.6, while $\delta_{1}=\delta_{3}=2.7$. The time-switching coefficient is equal to α = 0.5. For all analyzed cases, secrecy outage probability is lower in the case when the Nakagami-m fading parameter in the link to the legitimate receiver has higher values, i.e., fading severity is less significant. Moreover, secrecy performances are improving for lower values of path loss parameter *δ*_2_, as the path loss value is lower for the same link length and the corresponding SNR of the legitimate receiver is higher.

**Figure 9:**
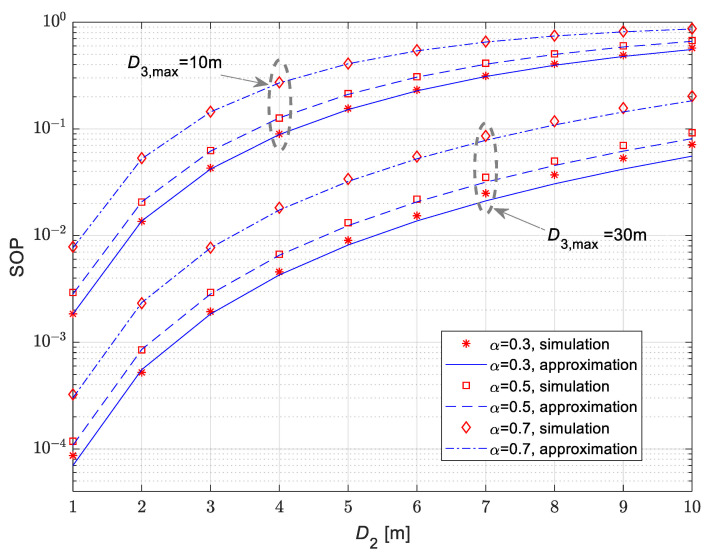
Secrecy outage probability in the case of fixed legitimate receiver for various time-switching ratios for wireless power transfer.

In Figure 9, the dependence of the secrecy outage probability vs. fixed position *D*_2_ of the legitimate receiver is presented for the case when the eavesdropper is moving in accordance with the 3D RWP model. The mean values of channel power gain are $\Omega_{10}=10\;\mathrm{dB}$, $\Omega_{20}=20\;\mathrm{dB}$ and $\Omega_{30}=10\;\mathrm{dB}$, while the corresponding Nakagami-*m* fading parameters are $m_{1}=m_{2}=m_{3}=2$. From the obtained results, it can be noticed that the secrecy outage probability increases with the rise of the fixed distance from the IoT transmitter to the legitimate receiver. Furthermore, the increase in the diameter of the 3D space, in which the eavesdropper is moving in accordance with the RWP model, leads to the decrease in secrecy outage probability values. The results are presented for different values of time-switching coefficients α of wireless power transfer. The increase of parameter α results in the increase of the secrecy outage probability. This effect is in accordance with the expectations, as the time dedicated to power transfer leads to a smaller part of each frame dedicated to information transfer and consequently to smaller secrecy capacity and increased secrecy outage probability.

Finally, based on the results presented in Figure 7, Figure 8 and Figure 9, it can be concluded that the approximate closed-form expression (34) accurately estimates secrecy outage probability values.

**Figure 10:**
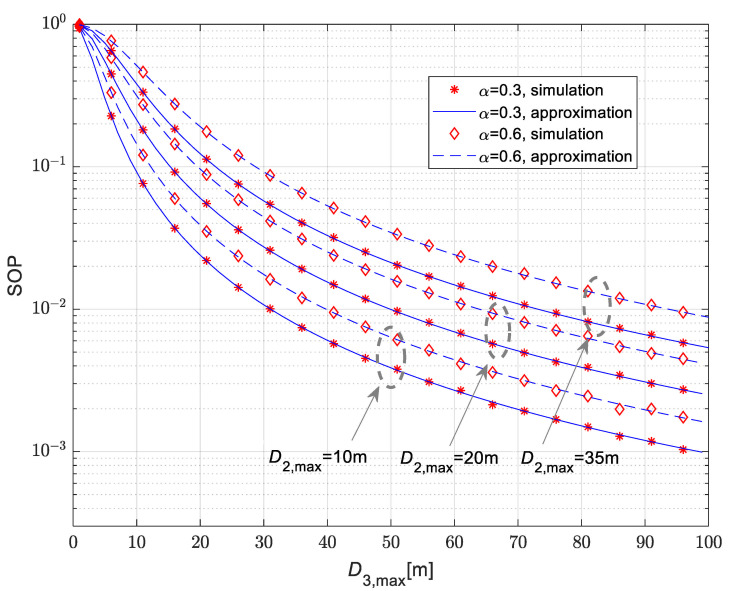
Secrecy outage probability vs. maximal distance of the legitimate receiver for the scenario when both legitimate receiver and eavesdropper are mobile.

The secrecy outage probability for the case when both the legitimate receiver and eavesdropper are moving is presented in Figure 10. The Nakagami-*m* fading parameters take non-integer values equal to $m_{1}=2.1$, $m_{2}=3.8$ and $m_{3}=1$. Secrecy performances are analyzed for the path-loss coefficients equaling $\delta_{1}=2.7$, $\delta_{2}=2.05$ and $\delta_{3}=3$. For the presented results, it is assumed that both nodes are moving in a plane in accordance with a 2D pattern. The secrecy outage probability decreases with the maximal distance between the IoT transmitter and the eavesdropper $D_{3,\max}$. Furthermore, the analyzed performance worsens for larger values of maximal distance between IoT transmitter and legitimate receiver, as it has a higher value for $D_{2,\max}=35\;m$ than in the case when $D_{2,\max}=10\;m$. The secrecy outage probability in this case also increases for a larger time-switching parameter α. For all analyzed cases, the results obtained by applying approximate closed-form expression (36) are in excellent agreement with the results obtained by the simulation method approach.

## 5. Conclusions

The secrecy outage performance is analyzed for the system where the IoT transmitter is energy-limited and supplied using a power beacon. The secrecy performances are analyzed for cases when either the legitimate receiver or eavesdropper is mobile and moving in accordance with the widely accepted RWP model. The accurate closed-form approximate expressions are derived for the case when the propagation environment can be modeled by Nakagami-*m* distribution and both integer and non-integer values of the Nakagami-*m* fading parameter. The closed-form approximate expressions for the secrecy outage probability are derived for the following important scenarios: (1) the legitimate receiver is fixed and the eavesdropper is mobile, (2) the legitimate receiver is mobile and the eavesdropper is fixed and (3) both the legitimate receiver and eavesdropper are mobile. The independently developed Monte Carlo simulation model corroborates the accuracy of the derived analytical expressions. The acquired numerical results show the impact of the system and channel parameters on the secrecy outage performances.

In all analyzed cases, the increase of the time-switching coefficient, which is proportional to the time dedicated to the wireless power transfer, leads to the increase in the secrecy outage probability. Further, obtained results demonstrate that secrecy performance declines with the increase of the maximal distance between the transmitter and the legitimate receiver, while improving with the rise of the maximal distance of the eavesdropper from the transmitter. On the one hand, when the eavesdropper is fixed, the best results are obtained in the case when the legitimate receiver randomly moves within the line pattern, while the worst results are obtained in the case when it moves within the sphere. On the other hand, in the case when the legitimate receiver has a fixed position, the secrecy performance improves with the increase of the number of dimensions within which the eavesdropper randomly moves.

The provided analysis presents the framework that can be useful for the design of future secure IoT systems positioned in inaccessible or hazardous areas, where data collecting from IoT sensors can be performed using units placed on mobile ground or aerial vehicles, while communication is endangered by eavesdroppers that can also be mobile. However, the analysis provided in this paper has limitations in some assumptions under which it was performed. First, although the Nakagami-*m* model is general and encompasses other propagation models as special cases, it does not cover all possible effects, such as shadowing. Second, multiple eavesdroppers can endanger the communication of the legitimate receiver and further degrade secrecy performances, compared to the case of a single eavesdropper. Also, ideal hardware characteristics are assumed, and characteristics deviation from the ideal ones can also degrade performances.

Finally, it is important to highlight that although the derived expression is approximate, its accuracy is demonstrated for a wide set of scenarios. Our future work will encompass the impact of shadowing on the system secrecy performances, as well as the extension to the case with multiple eavesdroppers.

## Figures and Tables

**Table 1:** Polynomial coefficients for the RWP model [28].

Dimension	*n*	*B_l_*	*β_l_*
1	2	[28]	[1, 2]
2	3	(1/73) [1, 2]	[1, 3, 5]
3	3	(1/72) [1, 3, 5]	[2, 4, 6]

## Data Availability

Data are contained within the article.
